# Do non-responders of a geriatric screening questionnaire face lower one-year survival compared to responders? A retrospective cohort study

**DOI:** 10.1007/s11136-026-04247-3

**Published:** 2026-05-03

**Authors:** Kathrine Hønholt Carlson, Lone Duval, Birgith Engelst Grove, Christina Bach Menzel, Claire Snyder, Jesper Ryg, Jonas Hermann Schrøder, Olalekan Lee Aiyegbusi, Regine Grytnes, Liv Marit Valen Schougaard

**Affiliations:** 1 AmbuFlex, Centre for Patient-reported Outcomes, Gødstrup Hospital, Herning, Denmark; 2 Department of Oncology, Gødstrup Hospital, Herning, Denmark; 3 Department of Clinical Medicine, Aarhus University, Aarhus, Denmark; 4 Departments of Medicine, Oncology, and Health Policy & Management, Johns Hopkins School of Medicine and Bloomberg School of Public Health, Baltimore, MD USA; 5 Geriatric Research and Clinical Evidence (GRACE), Department of Internal Medicine Geriatric Section, Copenhagen University Hospital – Herlev and Gentofte Hospital, Copenhagen, Denmark; 6 Department of Clinical Medicine, University of Copenhagen, Copenhagen, Denmark; 7 Geriatric Research Unit, Department of Clinical Research, University of Southern Denmark, Odense, Denmark; 8 Centre for Patient Reported Outcomes Research, Department of Applied Health Sciences, University of Birmingham, Birmingham, UK; 9 National Institute for Health and Care Research (NIHR), Birmingham Biomedical Research Centre, University of Birmingham, Birmingham, UK

**Keywords:** Patient-reported outcomes measures, Non-responding, Cancer, Survival

## Abstract

**Purpose:**

A geriatric screening questionnaire was implemented in 2020 at the Department of Oncology, Gødstrup Hospital, Denmark, to identify frailty and individualize cancer care for all patients aged ≥ 60 years. However, not all patients answered. The primary aim of this study was to investigate the association between response status to the geriatric screening questionnaire and one-year survival. A secondary aim was to compare one-year survival according to the responders’ frailty status.

**Methods:**

We conducted a retrospective cohort study including all patients with cancer aged ≥ 60 who were enrolled in the geriatric screening between August 2020 and October 2023. The survival curves for responders and non-responders and across the patients’ frailty status were visualized using Kaplan–Meier plots. The associations were analyzed using logistic regression adjusted for potential confounders.

**Results:**

In total, 702 patients were included. Among these, 28.1% were non-responders, 46.7% were classified as high risk of frailty, and 25.2% as low risk of frailty. The non-responders had the lowest survival, which declined rapidly in the first 100 days. Survival was similarly lower among non-responders and responders at high risk of frailty, compared to responders at low risk. When adjusted for cohabiting status, age, and cancer type, the odds ratio of survival among non-responders compared to responders was 0.63 (95%CI: 0.42;0.92).

**Conclusion:**

The non-responders had the lowest survival, comparable to patients at high risk of frailty among the responders. Their survival declined rapidly suggesting that non-response may signal vulnerability and warrant closer clinical attention or early supportive care.

**Supplementary Information:**

The online version contains supplementary material available at 10.1007/s11136-026-04247-3.

## Background

Life expectancy is increasing worldwide, resulting in a rise in the proportion of older individuals [1]. The number of new cancer cases is expected to increase by more than 200% in 2050 compared to 2018, and a large proportion will be diagnosed in older persons [2]. Functional status is an important aspect of health-related quality of life [3]. Older patients with cancer display a heterogeneous functional health status, ranging from frail to fit [4]. Frailty is associated with an increased risk of intolerance of cancer treatment [4]. Thus, treatment strategies for older patients must be carefully individualized to avoid both undertreatment and overtreatment [5–7]. The individualisation of care can be difficult. One reason is that older patients, especially those with frailty, are often underrepresented in cancer clinical trials, which limits the generalizability of the research to this population [6, 8, 9]. Another reason is that older patients often have polypharmacy, comorbidity, reduced organ function, and may lack social support, which complicates the treatment strategy [5, 9].

Traditionally, Eastern Cooperative Oncology Group (ECOG) performance status (PS) has been used to support individualization of the treatment strategy [5]. ECOG PS is a brief oncologist-reported health status assessment, ranging from 0 (fully active) to 5 (dead) [10]. Although poor PS is associated with chemotherapy intolerance, PS often appears normal despite underlying deficits that predict intolerance [11, 12]. An alternative method for evaluating the patient’s health status is comprehensive geriatric assessment (CGA), as recommended by the International Society of Geriatric Oncology [3, 13]. A systematic review found that CGA increases treatment completion, reduces toxicity, improves quality of life, promotes less intensive treatment, and supports care strategies, while no effect on survival has been found [14]. Using CGA for all patients is, however, time- and resource-intensive in daily clinical practice [15]. A more feasible solution is geriatric screening (GS) that can help oncologists identify frail patients in need of CGA [16]. Several validated tools exist, including the frequently used screening questionnaires the Vulnerable Elders Survey-13 (VES-13) and Geriatric-8 (G8) [16, 17].

Since 2020, an electronic patient-reported outcome (ePRO) solution using GS has been implemented at the Department of Oncology, Gødstrup Hospital, Denmark [18]. Referred patients are asked to complete an electronic version of the GS questionnaire prior to their initial consultation. The questionnaire includes VES-13, G8, and four single items regarding fall tendency and social support. The oncologists use the patients’ questionnaire responses in combination with past medical history, medication review, laboratory parameters, and disease characteristics to tailor the treatment to the patients’ health status [18]. Being a non-responder to the GS questionnaire may lead to a less individualized treatment plan and an increased risk of poor clinical outcomes including chemotherapy intolerance, over- or undertreatment, and lower survival [5–7]. However, poor clinical outcomes are likely influenced not only by suboptimal treatment but also by greater pre-treatment frailty among non-responders [4, 19, 20]. Research on questionnaire non-responders in oncology research projects has found that non-responders more often experience restrictions in activities of daily living, impaired mobility, cognitive decline, and are more frail, older, and face an increased risk of imminent death [19–22]. To the best of our knowledge, no studies have explored whether clinical outcomes, such as survival, differ among GS questionnaire responders and non-responders in a geriatric oncology patient population in a clinical setting.

The primary aim of this study was to investigate the association between GS questionnaire responders versus non-responders and one-year survival. We hypothesized that non-responders face lower survival than responders. A secondary aim was to compare one-year survival among GS questionnaire responders based on their frailty status, as determined by the GS questionnaire. We hypothesized that responders at high risk of frailty and non-responders would have lower survival than responders at low risk of frailty.

## Methods

### Setting and participants

Starting in 2020, the Department of Oncology at Gødstrup Hospital, Denmark performed a stepwise implementation of a GS-ePRO solution. The implementation started with selected cancer diagnoses and gradually included additional groups. The target population of the ePRO solution consists of patients aged ≥ 60 years with cancer who understand and speak Danish and are registered to e-Boks, Denmark’s national secure digital communication platform [18]. The implementation was successful, and all newly referred patients aged ≥ 60 years are instructed to complete an electronic GS questionnaire prior to their first consultation at the Department.

All patients enrolled in the GS ePRO solution at the Department between August 2020 and October 2023 were included in this study. The study population was identified in the AmbuFlex database, which contains all newly referred cancer patients aged ≥ 60 years at the oncology department [23]. All patients were followed for up to one year, or until death or emigration, whichever came first. This retrospective cohort study was conducted in accordance with the STROBE guidelines for reporting observational studies (Appendix 1) [24].

### The GS ePRO solution

The GS questionnaire includes 24 items based on the G8 and VES-13 as well as four single items regarding community-based home care, whether the respondent lived alone, and falls within the last six months [18]. The single items concerning community-based home care and cohabiting were inspired by the Geriatric Core Dataset (G-CODE) whereas the item on fall tendency was newly developed [25]. In the GS ePRO solution, a color-coded algorithm is used for risk stratification, whereby items are categorized according to frailty assessment: high risk of frailty (red), moderate risk (yellow), and low/none risk (green). Table 1 shows an overview of thresholds for the G8 and VES-13 scores as well as the single items included in the GS questionnaire. The G8 consists of eight items covering eight domains regarding nutritional status, weight loss, body mass index, physical function, psychological status, number of medications, self-perceived general health, and age [26, 27]. The items are summarized as a total score ranging from 0–17. A score ≤ 14 indicates increased risk of frailty [28]. The VES-13 consists of 13 main items covering four domains: physical function, functional disabilities, self-rated health, and age. The VES-13 uses conditional branching for five items; supplementary items are displayed if a patient reports symptoms. The items are summarized into a score ranging from 0–10 [29]. A score of ≥ 3 indicates frailty [29]. The combined use of the G8 and VES-13 is suitable for assessing the risk of frailty with a sensitivity of 91.4% (95%CI: 79.3;97.6) and specificity of 93.8% (95%CI:73.6; 99.6) [30]. Additionally, the four single items in the GS questionnaire are color-coded, corresponding to the risk of frailty. The color-code thresholds are based on prior literature and clinical expertise [18, 28, 29]. Oncologists have access to the patients’ questionnaire responses through a PRO-based graphical overview integrated into the Electronic Health Record (EHR) [18]. They assess the patients’ responses in combination with other relevant clinical data before the first consultation at the Department to tailor cancer care according to the patient’s frailty [18]. This specific GS ePRO solution was developed using the generic PRO-system AmbuFlex, which has been used to implement 82 PRO solutions for various patient groups in the Central Denmark Region and the Northern Denmark Region, including patients with cancer [23, 31].

**Table 1:**
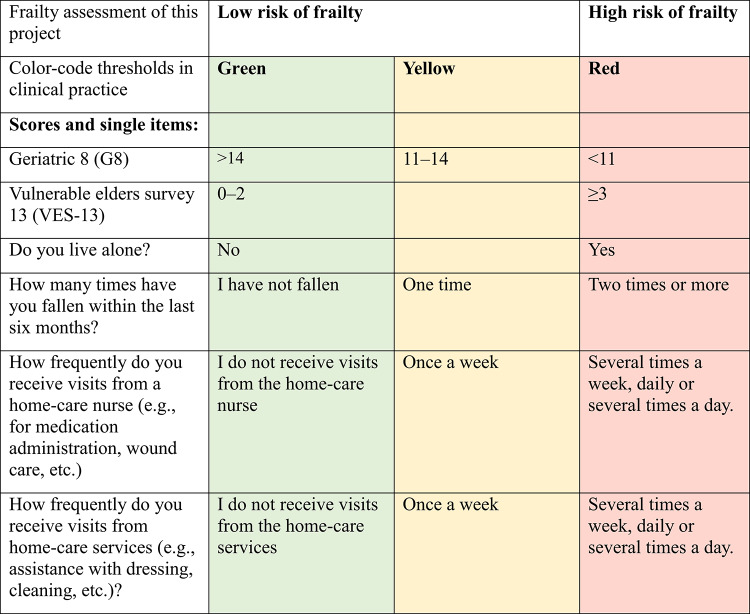
Color-code thresholds for the geriatric screening electronic patient-reported outcome (GS ePRO) solution in clinical practice and the frailty assessment in this project

### Data

The data in this study were register-based. Patient characteristics, exposure, and outcome variables were obtained from the EHR and the Central Denmark Region’s data warehouse which includes data from the AmbuFlex system [31] and the Danish National Patient Registry [32].

#### Exposure and outcome

Patients were categorized as responders if they completed at least one GS questionnaire item. Non-responders were defined as patients who did not complete any questionnaire items.

The results of the G8, VES-13, and the single items were combined into one overall frailty score based on the worst frailty assessment from either the G8 score, the VES-13 score, or the single items (Table 1). The VES-13 and G8 scores were only calculated if all items in each score were completed. Patients were assigned high risk of frailty if they had at least one item or score that was color-coded red. If the patient did not have any red items/scores but had at least one item or score that was color-coded yellow, they were assigned low risk of frailty. If they did not have any red or yellow items/scores, they were assigned no frailty. Due to the limited number of participants with no frailty, the “no frailty” and “low risk of frailty” groups were combined to ensure data confidentiality. Thus, the assessed frailty was categorized into two groups: low risk of frailty and high risk of frailty. The outcome was overall survival one year after the date for referral to the GS ePRO solution.

#### Patient characteristics

Information about sex, age, PS, cohabiting status, treatment intention, comorbidity, primary cancer diagnosis, and their responses from the GS were obtained.

Sex was a binary variable: “female” or “male”, and age was divided into three groups: 60–69, 70–79, or 80 +. PS was classified by the oncologist according to the ECOG method at the time of the diagnosis [10]. If the oncologist had registered that the patient was between two PS descriptions, the better PS was selected. Cohabiting status was divided into two categories: living alone and living with a partner. Treatment intention was dichotomized into palliative or adjuvant, as classified by the oncologist. Information about primary cancer was grouped according to ICD-10 cancer diagnosis. If fewer than 15 patients in total had a specific cancer type, it was grouped and classified as other specified cancers.

### Analytic approach

The distribution of patient characteristics were summarized for responders and non-responders, and by frailty status. The frequency of item non-response was examined according to frailty status. A chi-squared test, along with the corresponding p-value, was used to assess any statistically significant differences between the groups. Kaplan–Meier curves were used to visualize survival differences by questionnaire response status (responders vs. non-responders) and by frailty status. Due to complete follow-up data, non-proportional survival curves and a fixed one-year follow-up period, the association between questionnaire response status and survival was analyzed using logistic regression. Likewise, the association between frailty status and survival was analyzed through logistic regression. Potential confounders were identified through a literature search and clinical expertise, and Directed Acyclic Graphs (DAGs) were used to visualize which confounders to include in the logistic regression (21, 32–36) (Appendix 2). In the logistic regression comparing survival by response status, age, cohabiting status, and primary cancer were identified as potential confounders, while sex and primary cancer were identified in the logistic regression comparing survival by frailty status. Age and cohabiting status were excluded as potential confounders from the logistic regression comparing frailty status, since they are included in the GS questionnaire. PS was excluded as a potential confounder due to the risk of overadjustment, as it is an alternative method of frailty assessment.

### Ethics

The study was conducted in accordance with the Danish law on data protection and approved by the Danish Data Protection Agency (ref. number 1-16-02-63-24). Data collection from patients’ electronic records was approved internally in the Central Denmark Region (ref. number 1-45-70-46-24). According to Danish legislation, this project does not require approval by an external ethical committee, since it was based on registered data [33].

## Results

### Patient characteristics

A total of 702 patients were included in the study with a median age of 75 years (Interquartile range: 72–79) and 52.1% men. Of these, 197 (28.1%) patients were non-responders, 328 (46.7%) were responders classified as high risk of frailty, and 177 (25.2%) were responders classified as low risk of frailty. Complete item response was observed in 98.3% of responders at low risk of frailty, compared with 95.7% among responders at high risk (*p* = 0.07). Among responders with item non-response, between 2 and 23 of the 24 items were completed. Compared with responders, non-responders were older, had worse PS, were less likely to live with a partner, had lower prevalence of pulmonary cancer or urological cancer, and were less likely to survive after one year (Table 2). Compared with patients at low risk of frailty, non-responders were more often women, were older, had worse PS, were less frequently living with a partner, and had lower prevalence of urological cancer (Table 2).

**Table 2:** Characteristics of the 702 included patients in the geriatric screening solution at the department of oncology, Gødstrup hospital from 2020–2023, by questionnaire response status (responders vs. non-responders) and across frailty

	Total N = 702	Non-responders N = 197 (28.1%)	Responders N = 505 (71.9%)		
	Total N (%)	Non-responders total N (%)	Responders total N = 505 N (%)	Responders at high risk of frailty N = 328 N (%)	Responders at low risk of frailty N = 177 N (%)
**Sex**					*
Female	336 (47.9%)	95 (48.2)	241 (47.7%)	177 (54.0%)	64 (36.2%)
Male	366 (52.1%)	102 (51.8)	264 (52.3%)	151 (46.0%)	113 (53.8%)
**Age-group**			**	*	**
60–69	85 (12.1%)	13 (6.6%)	72 (14.3%)	43 (13.1%)	29 (16.4%)
70–79	470 (67.0%)	125 (63.4%)	345 (68.3%)	215 (65.6%)	130 (73.4%)
80+	147 (20.9%)	59 (30.0%)	88 (17.4%)	70 (21.3%)	18 (10.2%)
**Performance status** ^**a**^			**	*	**
0	141 (20.1%)	27 (13.7%)	114 (22.6%)	40 (12.2%)	74 (41.8%)
1	286 (40.7%)	66 (33.5%)	220 (43.6%)	137 (41.8%)	83 (46.9%)
2	160 (22.8%)	50 (25.4%)	110 (21.8%)	94 (28.7%)	16 (9.0%)
≥ 3	84 (12.0%)	40 (20.3%)	44 (8.7%)	43 (13.1%)	< 5
Missing	31 (4.4%)	14 (7.1%)	17 (3.4%)	14 (4.3%)	< 5
**Cohabiting status**			**	**	**
Living alone	199 (28.4%)	61 (31.0%)	138 (27.3%)	137 (41.8%)	< 5
Living with partner	479 (68.2%)	120 (60.9%)	359 (71.1%)	185 (56.4%)	174 (98.3%)
Missing	24 (3.4%)	16 (8.1%)	8 (1.6%)	6 (1.8%)	< 5
**Treatment intention**					
Adjuvant	186 (26.5%)	57 (28.9%)	129 (25.5%)	80 (24.4%)	< 50
Palliative	499(71.1%)	132 (67.0%)	367 (72.7%)	242 (73.8%)	125 (70.6%)
Missing	17 (2.4%)	8 (4.1%)	9 (1.8%)	6 (1.8%)	< 5
**Primary cancer**			*		**
Pulmonary	292 (41.6%)	76 (38.6%)	216 (42.8%)	150 (45.7%)	66 (37.3%)
Intestinal	208 (29.6%)	71 (36.0%)	137 (27.1%)	85 (25.9%)	52 (29.9%)
Urologic	55 (7.8%)	7 (3.6%)	48 (9.5%)	22 (6.7%)	26 (14.7%)
Pancreas	52 (7.4%)	13 (6.6%)	39 (7.7%)	30 (9.2%)	9 (5.1%)
Gynecologic	50 (7.1%)	12 (6.1%)	38 (7.5%)	23 (7.0%)	15 (8.5%)
Other specified cancers^b^	29 (4.1%)	12 (6.0%)	17 (3.4%)	13 (4.0%)	< 5
Unknown	16 (2.3%)	6 (3.1%)	10 (2.0%)	5 (1.5%)	< 10

IQR: Interquartile range, G8: Geriatric-8, VES-13: Vulnerable Elders Survey-13^a^ Performance status is based on the ECOG-method at the time of the diagnosis [10],^b^ Other specified cancer consists of Bone Cancer, Breast Cancer, Head and Neck Cancer, Multiple Myeloma, Stomach Cancer, and Thyroid Cancer. *p < 0.05, **p < 0.01, by chi-squared test compared with non-responders

### Survival for responders vs. non-responders

**Fig. 1:**
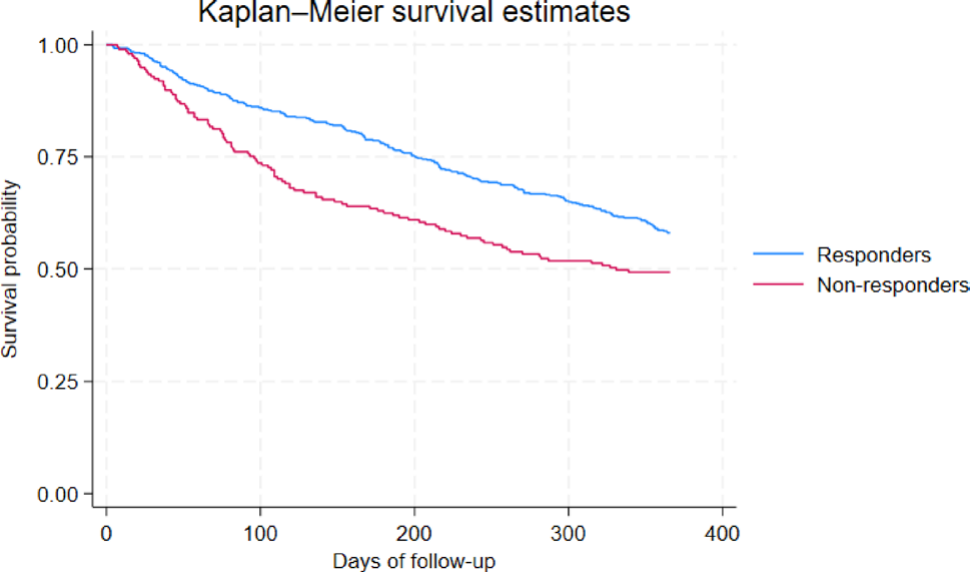
One-year survival for responders vs. non-responders

At one-year follow-up, the crude survival rate was 58.0% (*n* = 293) for the responders and 49.2% (*n* = 97) for the non-responders (Table 3). The Kaplan-Meier plot comparing one-year survival curves for responders versus non-responders showed that the survival curve for non-responders declined more rapidly than for responders in the first 100 days of follow-up, while the curves run approximately parallel from day 100 to day 300 (Fig. 1). Table 3  presents the results of logistic regression analyses of the one-year survival of responders compared to non-responders. The crude logistic regression showed that the odds of one-year survival were lower for non-responders compared to responders (OR: 0.70 (95% CI:0.50;0.98)). When adjusted for the confounders, the difference remained statistically significantly different (OR: 0.63 (95% CI: 0.42;0.92)) (Table 3 ).

**Table 3:** Odds ratio and 95% confidence interval (CI) of the logistic regression comparing one-year survival in patients responding to geriatric screening questionnaires compared to non-responders

	Total N	Survival N (%)	Crude OR (95% CI)	Adjusted ^a^ OR (95% CI)
Responders	505	293 (58.0%)	1 (ref)	1 (ref)
Non-responders	197	97 (49.2%)	0.70 (0.50;0.98)	0.63 (0.42;0.92)

^a^ Adjusted for cohabiting status, age, and cancer type

### Survival of responders according to frailty status vs. non-responders

**Fig. 2:**
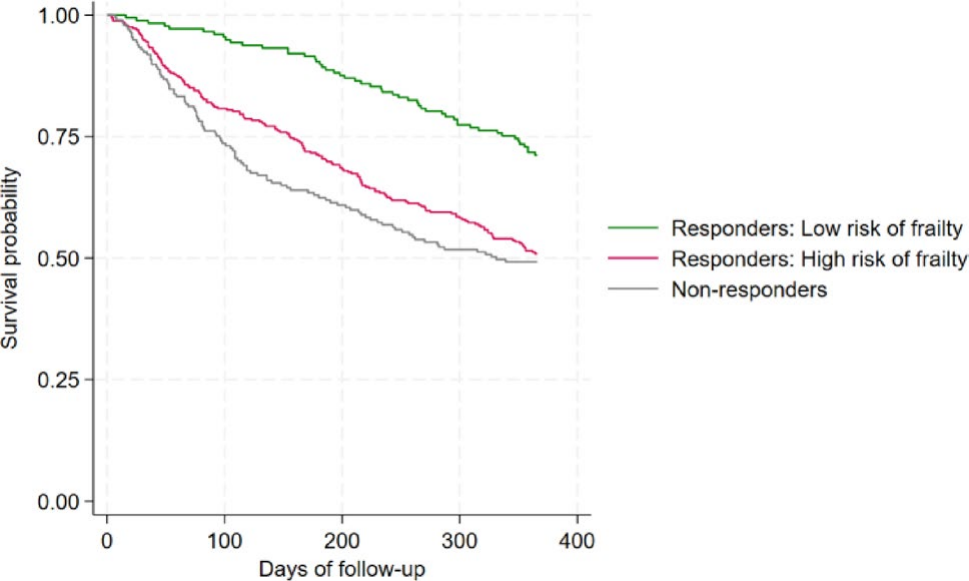
One-year survival for non-responders and for responders across frailty status

At one-year follow-up, the crude survival rate was 71.5% (*n* = 126) for the responders at low risk of frailty, 50.9% (*n* = 167) for the responders at high risk of frailty, and 49.2% (*n* = 97) for the non-responders (Table 4). Figure 2 presents the Kaplan-Meier curves comparing the one-year survival across frailty status. Patients at low risk of frailty had the highest survival probability, while non-responders and patients at high risk of frailty had similarly lower survival probabilities. The survival probability was lowest in the non-responders throughout the follow-up period. The results of the logistic regression are shown in Table 4. The crude logistic regression showed that the odds of survival were statistically significantly lower for non-responders and responders at high risk of frailty compared to responders at low risk of frailty. This tendency remained when adjusted for the potential confounders (Table 4).

**Table 4:** Odds ratio and 95% confidence interval (CI) of the logistic regression comparing one-year survival according to frailty score in patients responding to geriatric screening questionnaires compared to non-responders

	Total N	Survival N (%)	Crude OR (95% CI)	Adjusted ^a^ OR (95% CI)
Responders low risk of frailty	177	126 (71.5%)	1 (ref)	1 (ref)
Responders high risk of frailty	328	167 (50.9%)	0.42 (0.28;0.62)	0.43 (0.28;0.66)
Non-responders	197	97 (49.2%)	0.39 (0.26;0.60)	0.34 (0.21;0.55)

^a^ Adjusted for sex and cancer type

## Discussion

This study investigated survival in relation to response status of a GS questionnaire, used at the Department of Oncology, Gødstrup Hospital, and frailty status. This study showed that older patients with cancer who do not respond to a GS questionnaire have a lower survival probability at all times compared to those patients who respond to the questionnaire. Furthermore, when responders were divided into patients at low and high risk of frailty, survival of non-responders and responders at high risk of frailty were lower than those with low risk of frailty. Both Kaplan-Meier plots showed that within 100 days, the survival probability declined more rapidly among non-responders than responders, regardless of frailty status. After this period, the slopes of the curves became approximately parallel.

To the best of our knowledge, no prior study has investigated survival for non-responders to a geriatric screening tool in a clinical encounter. Several studies have investigated non-responders in research settings [19–21]. De Vries et al. found that questionnaire non-responders had higher odds of subsequent death, aligning with our results of reduced survival in non-responders [18]. De Rooij et al. showed that the survival probability of non-responders to a research questionnaire declined more rapidly initially after (non-)responding [20]. While both the findings of de Rooij et al. and our findings show that the survival curve eventually becomes parallel, the timing differs. In our study, the curves become approximately parallel after 100 days, whereas the results of de Rooij et al. find this pattern after two years. The observed discrepancy may be explained by the difference in timing of cancer diagnosis between the study populations. In de Rooij’s cohort, patients had been diagnosed 2–3 years before inclusion, while the patients in our study were newly diagnosed.

As shown in the DAGs in Appendix 2, the reduced survival among non-responders can be elucidated by two main explanations. Firstly, the GS ePRO solution provided the oncologists with more precise information about a patient’s frailty status. Thus, non-responders might have received less individualized supportive care as the oncologists lacked precise information about these patients, and this could explain the lower survival curves. Since a systematic review showed that the use of geriatric assessment, which is an expanded assessment of frailty, did not improve survival, the difference in survival found in this study is likely not caused solely by the use of GS [13]. Secondly, non-responders were more frail, which can increase the risk of lower survival. Research on questionnaires non-responders in oncology research projects has found that non-responders more often had more restrictions, had poorer PS, were more impaired, and were older [18–21]. Furthermore, de Vries et al. found that questionnaire non-response was associated with lower G8 score, possibly indicating greater frailty among non-responders [18]. This result echoes our findings, where non-responders were older and had poorer PS. Also, the survival curves of the non-responders were similar to the curves of the patients at high risk of frailty. Thus, the association might be a result of more frail patients, possibly in combination with less tailored care.

Results from a scoping review on reasons for non-participation in PRO solutions in clinical practice have shown that non-participation can be caused by several reasons [34]. Several of the studies included in the scoping review reported that older age, disease progression, health issues, and a lack of energy were reasons for non-participation, which is consistent with the hypothesis of frailty in our study [34]. However, other reasons for non-participation, not indicating frailty or poor health, are also common, such as technical issues, skepticism about the questionnaire, or data security concerns [34]. If the reasons for non-response in the current study are caused by a combination of health issues and other reasons unrelated to health, a stratified analysis of the impaired non-responders might show a more pronounced difference in survival. Oncology departments should be attentive to non-responders and recognize non-response as a potential indicator of frailty, as these patients have similar or worse survival than the most frail responders. The next step in implementation should be to introduce systematic geriatric assessments for non-responders to identify underlying frailty and provide timely supportive care.

Response rates in earlier studies range from 52–69%, which is lower compared to the 71.,9% in the GS ePRO solution from the Gødstrup Oncology Department [20–22]. This difference might be related to the GS ePRO solution being implemented in clinical practice, while earlier studies have investigated non-responders in research settings [20–22]. A scoping review of 14 studies on interventions to improve PRO response rates in oncology clinical practice showed that several interventions, such as real-time monitoring, clinical engagement, and reminders, can improve response rates [35]. Since non-responders potentially are more frail, they need individualized treatment strategies to avoid over- or undertreatment. Thus, further research on developing interventions to minimize non-response in this clinical setting should be conducted.

### Strengths and limitations

This study has several strengths. Firstly, no patients were lost to follow-up, and the data from the Central Denmark Region’s data warehouse are linked to the Danish National Patient Registry, which indicates overall high data quality and completeness [. Thus, the risk of information bias is low, and if an information bias exists, it is likely to be non-differential, which generally biases the results towards the null. Secondly, all patients are systematically referred to the GS ePRO solution, which means that they are not being selected based on individual clinicians’ subjective assessment of suitability [17]. Therefore, the risk of selection bias was reduced [36, 37]. Finally, as the study was conducted in a real-world clinical practice setting, the results are highly transferable to routine or similar healthcare environments.

The study also has some important limitations. Firstly, the stepwise implementation within the clinical setting meant that certain diagnostic groups were incorporated into the intervention at a later stage than others. As a result, the distribution of primary cancer diagnoses in the study population may differ from that typically observed in routine clinical practice, e.g., a relatively low number of breast cancer diagnoses. Secondly, although the overall implementation rate of the intervention is high, some oncologists do not consistently incorporate the GS into their clinical assessment. This incomplete uptake could dilute potential associations and lead to an underestimation of the true effect of the intervention. Thirdly, this was a single-center study, which may limit the generalizability of the findings to other settings or populations. Fourthly, we do not have any data regarding reasons for non-response. As the study is based on registry data, we were not able to explore participants’ motivations or barriers to participation, which might have been better understood through qualitative methods. Finally, analyses were conducted using the available sample, and the results should therefore be interpreted with consideration of the potential limitations in statistical precision.

## Conclusion

The non-responders had the lowest survival, comparable to responders at high risk of frailty. The survival probability declined more rapidly during the first 100 days among non-responders than responders, regardless of frailty status. These results highlight that oncology departments should be attentive to non-responders shortly after treatment initiation and recognize non-response as a potential indicator of frailty, as these patients have similar or worse survival than the most frail responders. Departments could implement comprehensive geriatric assessments for non-responders to identify underlying frailty and provide timely supportive care.

## Supplementary Information

Below is the link to the electronic supplementary material.

Supplementary Material 1

## Data Availability

No datasets were generated or analysed during the current study.
